# *Borrelia miyamotoi* Serology in a Clinical Population With Persistent Symptoms and Suspected Tick-Borne Illness

**DOI:** 10.3389/fmed.2020.567350

**Published:** 2020-10-27

**Authors:** Shannon L. Delaney, Lilly A. Murray, Claire E. Aasen, Clair E. Bennett, Ellen Brown, Brian A. Fallon

**Affiliations:** ^1^Lyme & Tick-Borne Diseases Research Center, Columbia University Irving Medical Center, New York, NY, United States; ^2^New York State Psychiatric Institute, Columbia University Irving Medical Center, New York, NY, United States

**Keywords:** *Borrelia miyamotoi* disease, relapsing fever borrelia, Lyme disease, serodiagnosis, post-treatment Lyme disease syndrome, borreliosis

## Abstract

Eighty-two patients seeking consultation for long-term sequalae after suspected tick-borne illness were consecutively tested for *Borrelia miyamotoi* antibodies using a recombinant glycerophosphodiester phosphodiesterase (GlpQ) enzyme immunoassay. Twenty-one of the 82 patients (26%) tested positive on the GlpQ IgG ELISA. Nearly all of the patients (98%) had no prior *B. miyamotoi* testing, indicating that clinicians rarely test for this emerging tick-borne pathogen. Compared to patients who solely tested positive for Lyme disease antibodies, patients with *B. miyamotoi* antibodies presented with significantly more sleepiness and pain. A prospective study is needed to ascertain the relationship between the presence of *B. miyamotoi* antibodies and persistent symptoms.

## Introduction

*Borrelia miyamotoi* is a relapsing fever spirochetal bacterium first identified in Japan in 1994 (1). The first human cases were reported in Russia in 2011 (2), and in the Northeastern United States in 2013 (3). *B. miyamotoi* is transmitted by the same hard-bodied ticks (*Ixodes* species) that are vectors of *Borrelia burgdorferi* (3, 4).

*B. miyamotoi* infection is clinically similar to Lyme disease (5) with manifestations of fever, fatigue, headache, myalgia, chills, and nausea (2, 4, 6, 7). Like Lyme disease, *B. miyamotoi* infection can lead to significant neurologic complications (5). However, unlike Lyme disease, erythema migrans rash, and arthralgias are uncommon (2). Overall, patients with acute *B. miyamotoi* infection often present with more severe symptoms, especially headaches and fever, than patients with acute Lyme disease (2). Meningoencephalitis can also occur in immunocompromised patients (2, 4). Relapsing fevers occur in a subset of patients with *B. miyamotoi* disease (2, 5). Like Lyme disease, *B. miyamotoi* disease is treated with 2–4 weeks of antimicrobial therapy—most often doxycycline or amoxicillin (2, 8, 9).

Polymerase chain reaction (PCR) analysis is used to confirm acute *B. miyamotoi* infection (9). Seroconversion is assessed using the glycerophosphodiester phosphodiesterase (GlpQ) enzyme immunoassay; IgM antibodies have been found to be reactive between 11 and 20 days after disease onset, and IgG antibodies reactive 21–50 days after disease onset (4). The GlpQ protein is present among all relapsing fever spirochetes, but absent in *B. burgdorferi* (4, 10). PCR positivity rates have ranged from 17% among patients hospitalized for acute infection with suspected tick-borne disease in Russia (2), to 0.7–0.8% (3, 6) among acutely ill patients in the Northeastern United States. A recent study in France showed that 43 of 824 patients (5.22%) with polymorphic signs and symptoms and suspected tick-borne illness were PCR positive for *B. miyamotoi* (5). Studies in North America using the GlpQ assay among patients with presumed tick-borne infection have revealed antibody seropositivity rates ranging from 3 to 21% Northeast (11), Canada (12), and California (13).

Although persistent neurocognitive and musculoskeletal complaints have been widely described among a subset of patients with Lyme disease (11), no research to date has investigated whether persistent or atypical symptoms can occur after *B. miyamotoi* infection (7). It is also unknown what proportion of patients in Lyme-endemic areas experience symptoms suggestive of tick-borne illness, but have negative diagnostic tests for Lyme disease and therefore do not receive antimicrobial treatment. This study is the first to investigate whether patients with chronic symptoms seeking consultation for suspected tick-borne illness show evidence of prior exposure to *B. miyamotoi*. In this report, we compare the prevalence and clinical characteristics of patients with positive serology to *B. miyamotoi* to those with *B. burgdorferi*.

## Methods

This study included 82 patients consecutively screened for *B. miyamotoi* and *B. burgdorferi* antibodies as part of a clinical workup for tick-borne illness at the Columbia University Irving Medical Center from June 2017 to October 2018. Chart review was approved by the New York State Psychiatric Institute IRB. Clinic patients were seeking a second opinion to determine whether their persistent polymorphic symptoms were attributable to tick-borne infection. Patients frequently endorsed chronic symptoms of fatigue, pain, neurocognitive, and psychiatric problems.

Evaluations consisted of comprehensive physician assessments, serologic testing, and questionnaires. The GLP-Q assay was an indirect EIA for detection of antibody to *B. miyamotoi*, performed at Imugen, Inc. in Norwood, MA. As previously reported, they use the GlpQ gene sequence from B. miyamotoi (GenBank accession number AY368276) as the basis for cloning and expression as a 38-kDa recombinant protein (rGlpQ) (6). *B. burgdorferi* antibodies were assessed using the C6 peptide ELISA and IgM and IgG western immunoblot. Patients were designated *B. miyamotoi* positive based on IgG or IgM GlpQ seroreactivity (defined as >1 to the value calculated for the highest result on the standard curve). Patients were designated Lyme-positive if they met 2017 CDC surveillance criteria for definite or probable Lyme disease, having an EM skin lesion or multisystem clinical symptoms with at least 5 positive IgG bands on the Western blot.

Patients rated their symptoms using the General Symptom Questionnaire-30 (GSQ-30), a measure specifically developed to assess multisystem symptom burden in patients with early Lyme disease and post-treatment Lyme disease syndrome (12). Patients also completed the Beck Depression Inventory-II, Cognitive Failures Questionnaire, Fatigue Severity Scale, Epworth Sleepiness Scale, McGill VAS Pain Scale, and Zung Anxiety Scale. Mann-Whitney *U* tests were conducted to compare *B. miyamotoi*-positive and *B. burgdorferi*-positive patients on these measures.

## Results

Of the 82 patients, 21 (26%) tested positive for *B. miyamotoi* by anti-GlpQ ELISA; all were IgG positive and IgM negative. Of these 21 patients, five also met CDC surveillance criteria for definite or probable Lyme disease. Of the 61 patients who tested negative for *B. miyamotoi*, 22 met criteria for definite or probable Lyme disease. The remaining 39 patients tested negative for both *B. miyamotoi* and *B. burgdorferi* antibodies.

The *B. miyamotoi*-positive group reported significantly more sleepiness on the Epworth Sleepiness Scale (*Md* = 9 vs. 4, *U* = 42.00, *z* = −2.51, *p* = 0.01) and significantly more pain on the VAS (*Md* = 5.80 vs. 2.70, *U* = 58.00, *z* = −2.02, *p* = 0.04) than the group with probable or definite Lyme disease. On the total score and individual item level, there were no significant between-groups differences on the GSQ-30, but the *B. miyamotoi*-positive group did endorse being bothered more by headaches than the *B. burgdorferi*-positive group (*Md* = 3 vs. 2 at a trend level, *U* = 52.50, *z* = −1.79, *p* = 0.07).

Data for *B. miyamotoi*-positive patients is presented in Supplementary Table 1. Mean age was 34 years. Eight of 21 (38%) reported hospitalization (seven medical and one psychiatric) since symptom onset, three for cardiac and two for neurologic abnormalities. All 21 received prior antibiotic treatment, of whom 20 received at least 2 weeks of doxycycline or amoxicillin. Sixteen of the 21 patients lived in the Northeast/Mid-Atlantic USA. Of the remaining five patients, two lived in California, two lived in Florida, and one lived in Illinois. Of the 82 patients in the study, 80 (98%) had not been previously tested for *B. miyamotoi* infection. Among the 21 patients positive on the GlpQ ELISA, 18 were also tested using the Lyme C6 ELISA and none were positive.

## Discussion

This is the first study to investigate the presence of *B. miyamotoi* antibodies in a clinical population experiencing persistent symptoms and suspected tick-borne illness. We found a high rate of *B. miyamotoi* GlpQ IgG antibody seropositivity (26%) among our patients seeking consultation for suspected tick-borne illness. This is a novel finding and higher than the seropositivity rates of 3–21% previously reported in the literature (13–15). There are likely many factors that may contribute to this finding. Firstly, *B. miyamotoi* is a common co-infection found in ticks (16). Given that *B. miyamotoi* is found 10 times less frequently in ticks than *B. burgdorferi* (17); we should still suspect at least 30,000 cases of *B. miyamotoi* disease in the US, compared to 300,000 presumed yearly cases of Lyme disease in the US (18). Secondly, unlike *B. burgdorferi, B. miyamotoi* can be transmitted via transovarial transmission, directly from adult tick to offspring, such that larval ticks can transmit infection as well as later stages (19). Thirdly, *B. miyamotoi* transmission from tick to human also occurs more quickly than *B. burgdorferi* transmission, the former occurring within 24 h of tick attachment, and the latter between 48 and 72 h (20). *B. miyamotoi* has been found in the midgut and salivary glands of both *Ixodes scapularis* and *Ixodes ricinis* ticks, likely contributing to faster transmission rates (21, 22). Lastly, it is possible that our clinical population is enriched for prior *B. miyamotoi* infection, as many patients presented with chronic symptoms and exposure to Lyme-endemic areas, but without erythema migrans rashes or positive Lyme serologic tests.

Our antibody assay detected the GlpQ antigen. GlpQ is found in other relapsing fever spirochetes throughout the world. In the United States, there are three primary species of relapsing fever spirochetes transmitted by soft ticks: *B. hermsii, B. parkeri, and B. turicatae*. Of the three*, B. hermsii* is the most common and is predominately found in the forested mountainous regions of the western United States (23). Our population was predominantly from the northeastern United States (16 of 21 *B. miyamotoi* positive patients), so it is unlikely that this finding could be due to a cross-reactivity with these soft-tick relapsing fevers. However, given that we did not obtain detailed travel histories, we cannot exclude the possibility of cross-reactivity. Furthermore, relapsing fever infection transmitted through soft ticks is thought to be rare in the United States; between the years of 1990 and 2011, only 504 cases of tick-borne relapsing fever were reported in the western United States (24).

Another notable finding from this study is that nearly all patients had not been previously tested for *B. miyamotoi* despite histories of tick exposure and subsequent symptoms and negative Lyme disease tests. Furthermore, many patients reported that their clinicians dismissed the possibility of tick-borne illness both at the onset and during the course of their illness and attributed symptoms to psychological stress. This underscores the need for more widespread clinician awareness of *B. miyamotoi* disease, especially because unlike Lyme disease, *B. miyamotoi* infection does not commonly present with an obvious pathognomonic sign, such as erythema migrans rash.

Our high seropositivity rate in this clinical sample, coupled with lack of prior testing, represents a significant public health concern. In Lyme disease, delayed diagnosis and treatment is associated with prolonged symptoms (25). The same may prove true for *B. miyamotoi* disease. Our study raises the research question of whether *B. miyamotoi* infection can lead to chronic symptoms. To decrease the risk of prolonged morbidity, patients with an acute onset of multi-system symptoms suggestive of Lyme disease or other tick-borne illnesses should also be tested for *B. miyamotoi* infection. While the PCR assay is optimal for patient assessment in acute infection, the antibody assay helps to clarify who has been previously infected; this is particularly relevant to those whose symptoms have been present for months or longer. While a positive GlpQ IgG does not confirm current infection, it strongly supports prior infection and would provide a measure of validation and relief for patients whose symptoms suggest tick-borne disease, but all laboratory tests were negative.

Our preliminary data indicate considerable overlap in the post-treatment symptom profile of patients with *B. miyamotoi* and *B. burgdorferi* antibodies. However, our patients with *B. miyamotoi* antibodies reported more sleepiness and pain than our patients with *B. burgdorferi* antibodies. Intriguingly, none of our *B. miyamotoi*-positive patients were also *B. burgdorferi*-positive on the C6 peptide ELISA. This was unexpected given prior research showing a high Lyme C6 positivity rate (91.7%) among patients with PCR-positive *B. miyamotoi* infection (26). This discrepancy may be due to the fact that our patients with chronic symptoms had all been previously treated with antibiotics for possible Lyme disease, while patients in prior studies were tested prior to treatment when they were acutely ill with active *B. miyamotoi* infection.

A limitation of this study is that we cannot verify a causal relationship between prior *B. miyamotoi* infection and chronic, non-specific symptoms in our patient sample. Because it is difficult to determine the date of infection in an illness that does not usually present with an obvious external sign, such as a rash, it is unclear whether patients' ongoing symptoms were temporally related to *B. miyamotoi* infection. This complexity is compounded by the variable disease presentation of relapsing fever borreliosis (27). Moreover, it is unknown how long *B. miyamotoi* antibodies persist after infection. Thus, to establish causality between infection and symptoms, a prospective longitudinal study following a larger sample of patients with acute *B. miyamotoi* disease is needed. Second, because our study relied on the GlpQ antibody assay rather than on a PCR assay, we cannot determine whether current infection was present at the time of our evaluation. Third, our sample size was relatively small.

Our study demonstrates that *B. miyamotoi* disease is rarely considered in the differential diagnosis of tick-borne illness. Our findings suggest that all patients presenting with symptoms indicative of a potential tick-borne illness in the absence of an erythema migrans rash should be tested for *B. miyamotoi* disease, using both PCR and antibody-based testing. Identifying *B. miyamotoi* seropositivity among patients suffering from chronic illness represents a significant finding warranting further investigation. Our findings raise the question of whether *B. miyamotoi* infection can lead to post-treatment sequelae, similar to Lyme disease. Given that *B. miyamotoi* disease is an emerging tick-borne illness, further basic science research and *in-vitro* models are needed to clarify the mechanisms and optimal treatment of *B. miyamotoi* disease (22).

## Data Availability Statement

The raw data supporting the conclusions of this article will be made available by the authors, without undue reservation.

## Ethics Statement

The studies involving human participants were reviewed and approved by New York State Psychiatric Institute. Written informed consent to participate in research was provided by the participants or their legal guardian/next of kin.

## Author Contributions

SD and BF contributed to the study design and clinical consultation. SD and LM contributed to the writing of the manuscript and BF contributed to the editing of the manuscript. SD, CA, CB, EB, LM, and BF contributed to the analysis of data and CB performed the statistical analysis. All authors contributed to the article and approved the submitted version.

## Conflict of Interest

The authors declare that the research was conducted in the absence of any commercial or financial relationships that could be construed as a potential conflict of interest.
