# Supplementary material for: Borrelia miyamotoi Serology in a Clinical Population With Persistent Symptoms and Suspected Tick-Borne Illness
Source: Front Med (Lausanne). 2020 Oct 27;7:567350. doi: 10.3389/fmed.2020.567350 (PMC7652925; doi:10.3389/fmed.2020.567350)
Supplement: Supplementary file 1 [file Table_1.DOCX]

**Supplemental Table 1**. Characteristics of Patients Positive by GlpQ Enzyme Immunoassay

| **Age (y)** | **Sex** | **GlpQ Antibody Titer** | **Signs and Symptoms During Course of Illness** | **Rash** | **CDC Lyme Classification** | **Duration of Symptoms** |
| --- | --- | --- | --- | --- | --- | --- |
| 47 | F | 1.1 | Headache, tinnitus, palpitations, night sweats, paresthesia | No | - | 2 years |
| 29 | F | 1.3 | Vestibular dysfunction, fatigue, arthralgias | No | - | 6 years |
| 37 | F | 2.4 | Tinnitus, dizziness, hearing loss, cognitive dysfunction, paresthesia, brain fog, joint/muscle pain, anxiety, “crawling sensations” | Yes, non-EM | - | 1 year |
| 30 | M | 1.3 | Fatigue, brain fog, joint/muscle pain | No | - | 2 years |
| 24 | F | 5.2 | Fatigue, brain fog, arthralgias, POTS | Self-reported EM | - | >10 years |
| 19 | F | 5.6 | Spine, limb and joint pain; cognitive dysfunction, headache | Yes, indeterminate | - | 3 years |
| 27 | M | 2.7 | Headache, neck & shoulder pain, cognitive slowing | No | - | 20 months |
| 32 | F | 1.8 | Brain fog, fatigue, myalgias, depression, visual changes | EM x 2 | Confirmed | 21 years |
| 52 | F | 2.3 | Myalgias, arthralgias, fatigue, anxiety, depressed mood, cognitive dysfunction | No | - | 2 years |
| 70 | M | 1.8 | Cognitive complaints, a-fib, headache, joint pain, initial high fever | EM | Confirmed | 5 years |
| 17 | F | 2.9 | Fatigue, neurocognitive impairment, OCD-like symptoms, POTS | Yes, non-EM | - | 2 years |
| 60 | M | 5.2 | Facial palsy, arthralgias, polyneuropathy, cognitive symptoms | EM | Confirmed | 2 years |
| 49 | F | 1.5 | Brain fog, fatigue, hyperacusis, depressed mood | EM x 3 | Confirmed | 4 years |
| 10 | M | 8.5 | Fever, headaches, dizziness, nausea, cognitive problems, personality changes, AV block | No | Probable | 1 year |
| 37 | M | 8.4 | Arthralgias, myalgias, cognitive dysfunction, neuropathy, fatigue | No | - | 18 months |
| 38 | F | 10.5 | Headaches, muscle pains, fatigue | Yes, non-EM | - | 18 months |
| 15 | M | 2.1 | Fatigue, cognitive problems, anxiety | No | - | 3 years |
| 34 | F | 3.8 | Myalgias, weakness, fatigue, brain fog, joint and chest pain | Self-reported EM | - | 1 year |
| 15 | F | 6.3 | Flu-like illness, heat intolerance, brain fog, myalgias, constipation/diarrhea | No | - | 18 months |
| 39 | F | 1.6 | Fatigue, cognitive problems and pain | No | - | 4 years |
| 28 | F | 2.3 | Myalgias, pleuritic chest pain, fatigue, headache, cognitive problems | No | - | 8 years |
